# High-Fat Diet Induces Apoptosis of Hypothalamic Neurons

**DOI:** 10.1371/journal.pone.0005045

**Published:** 2009-04-02

**Authors:** Juliana C. Moraes, Andressa Coope, Joseane Morari, Dennys E. Cintra, Erika A. Roman, José R. Pauli, Talita Romanatto, José B. Carvalheira, Alexandre L. R. Oliveira, Mario J. Saad, Licio A. Velloso

**Affiliations:** 1 Department of Internal Medicine, University of Campinas, Campinas, Brazil; 2 Department of Anatomy, University of Campinas, Campinas, Brazil; University of Texas Health Science Center, United States of America

## Abstract

Consumption of dietary fats is amongst the most important environmental factors leading to obesity. In rodents, the consumption of fat-rich diets blunts leptin and insulin anorexigenic signaling in the hypothalamus by a mechanism dependent on the *in situ* activation of inflammation. Since inflammatory signal transduction can lead to the activation of apoptotic signaling pathways, we evaluated the effect of high-fat feeding on the induction of apoptosis of hypothalamic cells. Here, we show that consumption of dietary fats induce apoptosis of neurons and a reduction of synaptic inputs in the arcuate nucleus and lateral hypothalamus. This effect is dependent upon diet composition, and not on caloric intake, since pair-feeding is not sufficient to reduce the expression of apoptotic markers. The presence of an intact TLR4 receptor, protects cells from further apoptotic signals. In diet-induced inflammation of the hypothalamus, TLR4 exerts a dual function, on one side activating pro-inflammatory pathways that play a central role in the development of resistance to leptin and insulin, and on the other side restraining further damage by controlling the apoptotic activity.

## Introduction

Obesity results from an imbalance between caloric intake and energy expenditure. Changes in lifestyle, resulting in increased consumption of dietary fats and reduced physical activity have contributed to the worldwide obesity epidemic [1]. Recent studies have shown that consumption of dietary fats promotes hypothalamic resistance to the main anorexigenic hormones, leptin and insulin, leading to the progressive loss of the balance between food intake and thermogenesis and, therefore, resulting in body mass gain [2]–[4]. The functional resistance to leptin and insulin in the hypothalamus is a consequence of diet-induced activation of inflammatory signaling, specifically in this site of the brain, which leads to the molecular impairment of leptin and insulin signal transduction by at least four distinct mechanisms; induction of suppressor of cytokine signaling-3 (SOCS3) expression [5], activation of c-Jun N-terminal kinase (JNK) and I kappa kinase (IKK) [3], and induction of protein tyrosine phosphatase 1B (PTP1B) [6].

Inflammatory and apoptotic pathways are tightly connected and subtle changes in some of the respective determining factors can swing the balance towards one or another outcome [7]. For example, the activation of signal transduction pathways by cytokines such as TNF-α and IL-1β can lead either to pro-, or anti-apoptotic effects in addition to their classical inflammatory activity [8], [9].

Since the balance between survival and loss of hypothalamic neurons may have an impact on the coordinated control of feeding and thermogenesis [10], [11], we decided to evaluate whether the consumption of high amounts of dietary fat can induce apoptosis of cells in this anatomical region. Our results show that neuronal apoptosis is induced by the fat-rich diet and that the presence of a functional TLR4 receptor protects hypothalamic cells from apoptotic damage.

## Materials and Methods

### Antibodies, chemicals and buffers

The reagents for SDS–polyacrylamide gel electrophoresis and immunoblotting were from Bio-Rad (Richmond, CA, USA). HEPES, phenylmethylsulfonyl fluoride (PMSF), aprotinin, dithiothreitol (DTT), Triton X-100, Tween 20, glycerol and bovine serum albumin (fraction V) were from Sigma (St. Louis, MO, USA). The reagents for chemiluminescence labeling of proteins in blots were from Amersham (Aylesbury, UK). Antibodies against SOCS3 (rabbit polyclonal, sc-9023), PARP (rabbit polyclonal, sc-7150), Bcl2 (rabbit polyclonal, sc-492), phospho-Bad (pBad) (goat polyclonal, sc-7999), Bax (rabbit polyclonal, sc-493), Apaf1 (goat polyclonal, sc-26685), caspase-9 (rabbit polyclonal, sc-7885), FADD (rabbit polyclonal, sc-5559), caspase-8/p20 (rabbit polyclonal, sc-7890), neuromedin-N (NeuN) (goat polyclonal, sc-7593), phospho-PERK (pPERK) (rabbit polyclonal, sc-32577), PERK (rabbit polyclonal, sc-13073), phospho-eIF2α (peIF2α) (rabbit polyclonal, sc-12412), eIF2α (rabbit polyclonal, sc-11386), IκB (rabbit polyclonal, sc-1643), NFκB/p50 (rabbit polyclonal, sc-7178), Myd88 (rabbit polyclonal, sc-11356), phospho-JNK (pJNK) (rabbit polyclonal, sc-12882), phospho-IKK (pIKK) (rabbit polyclonal, sc-23470), POMC (rabbit polyclonal, sc-20148), AgRP (rabbit polyclonal, sc-18634), TLR4 (rabbit polyclonal, sc-13591), F4/80 (rabbit polyclonal, sc-25830), and FITC or rodhamin conjugated goat and rabbit antibodies were from Santa Cruz Biotechnology (Santa Cruz, CA, USA). The annexin V FITC was produced by the Laboratory of Molecular and Cellular Biology at the Institute of Biomedical Sciences, State University of São Paulo, Brazil. The synaptophysin (rabbit polyclonal, A0010) antibody was from DAKO (Glostrup, Denmark). The caspase-3 antibody was form Cell Signaling (Danvers, MA, USA). The kit for detecting apoptosis by the TUNEL assay was from Upstate Cell Signaling Solutions (Temecula, CA, USA). Chemicals for real-time PCR were from Invitrogen (Carlsbad, CA, USA) and Applied Biosystems (Foster City, CA, USA).

### Experimental model and feeding protocols

For most of the experiments, eight-week-old (280–300 g) male Wistar rats were employed. In addition, some experiments were performed with recombinant mice with a loss-of-function mutation for the TLR4 gene (C3H/HeJ) and its respective control (C3H/HeN). In addition, some experiments were performed with male, 8-week old Swiss mice. Rats and Swiss mice were obtained from the University of Campinas Animal Breeding Center, while mutant mice were purchased from the Jackson Laboratory (Bar Harbor, Maine, USA). All the animals were handled according to the University guidelines for the use of animals in experimental studies and conform to the Guide for the Care and Use of Laboratory Animals, published by the US National Institutes of Health (NIH publication No. 85-23 revised 1996). All experimental protocols were approved by the University of Campinas Ethics Committee. The rats were always housed in individual cages and maintained on a 12 h-light/dark cycle. After random selection, rats and Swiss mice were submitted for eight weeks to a control or high-fat (HF) diet, as presented in Table 1. The access to diet and water was *ad libitum*. Some rats were submitted to caloric pair feeding for eight weeks. For that, 18.43 g of high-fat chow was offered every day, which provided a mean caloric intake of 4.3 kCal/g body weight, similar to the mean caloric intake of rats fed on the control diet. Eight-week old C3H/HeJ and C3H/HeN mice were randomly selected to either control or HF diet for 8 weeks. At the end of the experimental period, hypothalami were obtained for determination of protein expression and immunohistochemistry.

**Table 1:** Macronutrient composition of the diets.

	*Standard Chow*		*High-Fat Chow*	
	g%	kJ%	g%	kJ%
Protein	20	19	20	14
Carbohydrate	76	72	45	31
Saturated fat	4	9	35	55
kJ/g	17.5		24.1	

### Intracerebroventricular (icv) cannulation and analysis of leptin and insulin action in the hypothalamus

For the evaluation of leptin- and insulin-induced inhibition of food intake, rats were stereotaxically instrumented using a Stoelting stereotaxic apparatus, according to a previously described method [12], [13]. Coordinates were: anteroposterior, 0.2 mm/lateral, 1.5 mm/depth, 4.0 mm. Procedure efficiency was tested one week after cannulation by the evaluation of the drinking response elicited by icv angiotensin II [12]. After the experiments, cannula placement was also evaluated by histology. For determination of food intake, rats were food deprived for 6 h (from 12 to 18 h) and at 18 h were icv treated with insulin (2.0 µl, 10^−6^ M), leptin (2.0 µl, 10^−6^ M), or saline (2.0 µl). Food ingestion was determined over the next 12 h, during the dark cycle.

### Real-time PCR and PCR array

Hypothalamic total RNA was extracted using Trizol reagent (Life Technologies, Gaithersburg, MD, USA), according to the manufacturer's recommendations. Total RNA was rendered genomic DNA free by digestion with Rnase-free Dnase (RQ1, Promega, Madison, WI, USA). Samples obtained from three hypothalami from control and HF diet fed rats and Swiss mice were analyzed using a real-time PCR array (RT^2^ profiler PCR array rat apoptosis - SuperArray Bioscience Corp., Frederick, MD, USA) containing 84 apoptosis related genes, as shown in www.superarray.com/rt_pcr_product/HTML/PARN-012A.html. Real-time PCR analysis of gene expression was carried out in an ABI Prism 7500 sequence detection system (Applied Biosystems). The optimal concentration of cDNA and primers, as well as the maximum efficiency of amplification, were obtained through seven-point, 3-fold dilution curve analysis for each gene. Each PCR reaction contained 25–30 ng of reverse-transcribed cDNA (depending on the gene). Primers were purchased from Applied Biosystems and were: NPY, Rn00561681_m1; POMC, Rn00595020_m1; TLR4, Rn00569848_m1; F4/80, Rn01527631_m1 Emr1; GAPD, #4352338E, for rat; and, NPY, Mm00445771_m1; POMC, Mm00435874_m1; GAPD #4352339E for mouse. The PCR conditions were 2 min at 50°C; 10 min at 95°C, followed by 40 cycles at 95°C for 15 sec and 60°C for 60 sec. Real-time data were analyzed using the engine provided by Applied Biosystems.

### Tissue extraction, immunoprecipitation and immunoblotting

Rats were anesthetized and the hypothalami were dissected and immediately homogenized in solubilization buffer at 4°C [1% Triton X-100, 100 mM Tris–HCl (pH 7.4), 100 mM sodium pyrophosphate, 100 mM sodium fluoride, 10 mM EDTA, 10 mM sodium orthovanadate, 2.0 mM PMSF and 0.1 mg aprotinin/ml] with a Polytron PTA 20 S generator (model PT 10/35; Brinkmann Instruments, Westbury, NY, USA). Insoluble material was removed by centrifugation for 20 min at 9000×*g* in a 70.Ti rotor (Beckman, Fullerton, CA, USA) at 4°C. The protein concentration of the supernatants was determined by the Bradford dye binding method. Aliquots of the resulting supernatants containing 2.0 mg of total protein were used for immunoprecipitation with antibodies against TLR4, Myd88, FADD, Apaf1 and IκB at 4°C overnight, followed by SDS–PAGE, transfer to nitrocellulose membranes and blotting with anti-Myd88, TLR4, caspase-8, caspase-9 and NFκB, respectively. In direct immunoblot experiments, 0.2 mg of protein extracts were separated by SDS–PAGE, transferred to nitrocellulose membranes and blotted with anti-pJNK, PERK, pPERK, eIF2α, peIF2α, PARP, Bax, Bcl2 antibodies.

### Immunohistochemistry

Paraformaldehyde-fixed hypothalami were sectioned (5.0 µm) and used in regular single- or double-immunofluorescence staining using Bax, Bcl2, pBad, pJNK, pPERK, PERK, eIF2α, peIF2α, TLR4, F4/80, AgRP, POMC, NeuN, caspase-3 and synaptophysin antibodies, as previously described [14], [15]. Analysis and documentation of results were performed using a Leica FW 4500 B microscope. The hypothalami were sectioned from Bregma −1,6 to −4,2 mm. Every second of all consecutive section was analyzed. The anatomical correlations were made according to the landmarks given in a stereotaxic atlas [13]. The topographical views of the regions to be studied were obtained by hematoxylin-eosin staining of consecutive sections.

### Transmission electronic microscopy (TEM)

Hypothalami from rats fed on control and HF diets were dissected and maintained overnight at 4°C in fixative containing 2.5% glutaraldehyde and 0.5% paraformaldehyde in phosphate buffer (pH 7.4). The specimens were then trimmed, dehydrated and embedded in Durcupan (Fluka-Sigma-Aldrich, Seelze, Germany). Ultrathin sections from the arcuate nucleus were collected on formvar-coated copper grids, counterstained with uranyl acetate and lead citrate, and examined under a transmission electron microscope (Leo906, Zeiss) operated at 60 KV. Hypothalamus microenvironment was analyzed and neurons with normal and apoptotic morphology were identified and photographed using a digital image acquisition system (Morada, Zeiss).

### TUNEL

A terminal deoxynucleotidyl-transferase-mediated dUTP nick end-labeling (TUNEL) assay was used to identify double-stranded DNA fragmentation. Briefly, tissue slides were deparaffinized, treated with proteinase K (20 µg/ml) for 15 min at room temperature, and then quenched in 2.0% hydrogen peroxide. After rinsing in phosphate-buffered saline (PBS), pH 7.4, specimens were incubated in 1× equilibration buffer for 10–15 s. The slides were then incubated with terminal deoxynucleotidyl transferase (TdT) for 1.0 h at 37°C, blocked with stop/wash buffer, and incubated with peroxidase antibody for 30 min at room temperature. Negative control for the TUNEL assay was confirmed by staining the tissues in the same manner without primary antibody. Percentages of TUNEL-positive neurons were determined in at least 10 optical fields. Analysis was performed in five 5.0 µm non-consecutive sections from each hypothalamus.

### Statistical analysis

Data from the real-time PCR array were analyzed using the engine supplied by the manufacturer. Only mRNAs undergoing at least 2.0-fold variation from control were considered significantly modulated by the diet. Specific bands in immunoblots were scanned and submitted to a quantitative analysis using the Scion Image software (Scion Corp., Frederick, MD, USA). TUNEL positive cells, the apoptotic cells detected in low-magnification TEM and synaptophysin positive nerve terminals were field counted. All these parameters and the metabolic data obtained from the animals were analyzed by the Student's *t*-test.

## Results

**Figure 1:**
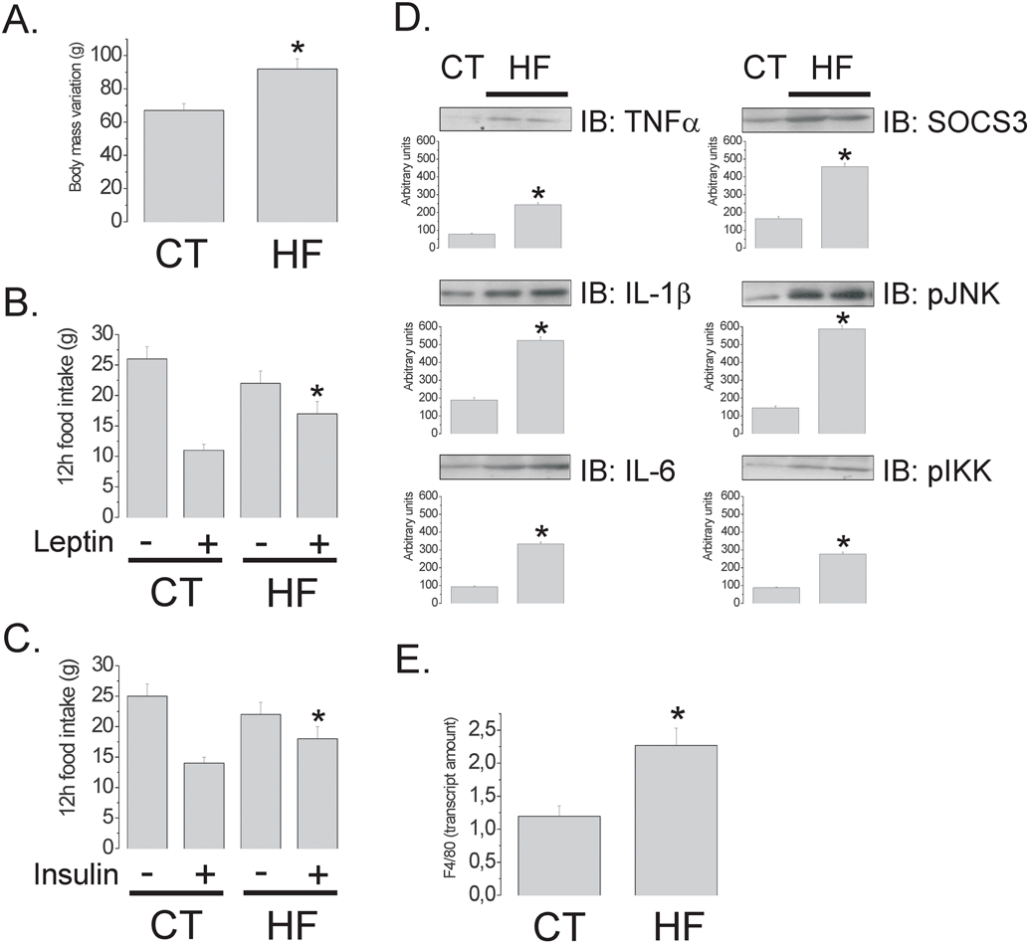
Leptin/insulin resistance and inflammatory markers in the hypothalamus of rats fed on high-fat diet. (A) Body mass variation (g) of Wistar rats fed on control (CT) or high-fat (HF) diets for 8 w. (B–C) Twelve hours spontaneous food intake (g) of Wistar rats fed on CT or HF diets for 8 w and treated icv with a single dose (2.0 µl) of saline (−), leptin (+, in B) or insulin (+, in C). (D) Immunoblots (IB) of hypothalamic protein extracts obtained from Wistar rats fed on CT or HF diets. (E) Real-time PCR analysis of F4/80 transcript amount in samples obtained from the hypothalami of Wistar rats fed on CT or HF diets. In all experiments n = 5. In A, D and E, *p<0.05 *vs.* CT, values are means±SEM; in B and C, *p<0.05 *vs.* CT+, values are means±SEM.

**Figure 2:**
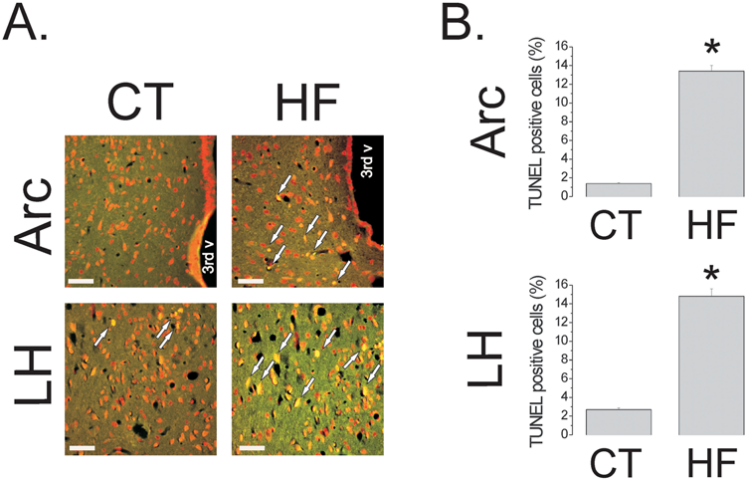
TUNEL assay depicts apoptosis in the hypothalamus of rats fed on high-fat diet. (A) Representative microphotographs of DNA fragmentation detection by TUNEL (stained in yellow) in samples from arcuate (Arc) and lateral hypothalamic (LH) nuclei, from Wistar rats fed on control (CT) or high-fat (HF) diets; the arrows indicate TUNEL positive cells. (B–C) TUNEL positive cells in Arc (B) and LH (C) are expressed as % of total cells per field. In all experiments n = 5. In A, magnification, *×200* (scale bar, 20 µm). In B and C, *p<0.05 *vs.* CT, values are means±SEM.

**Figure 3:**
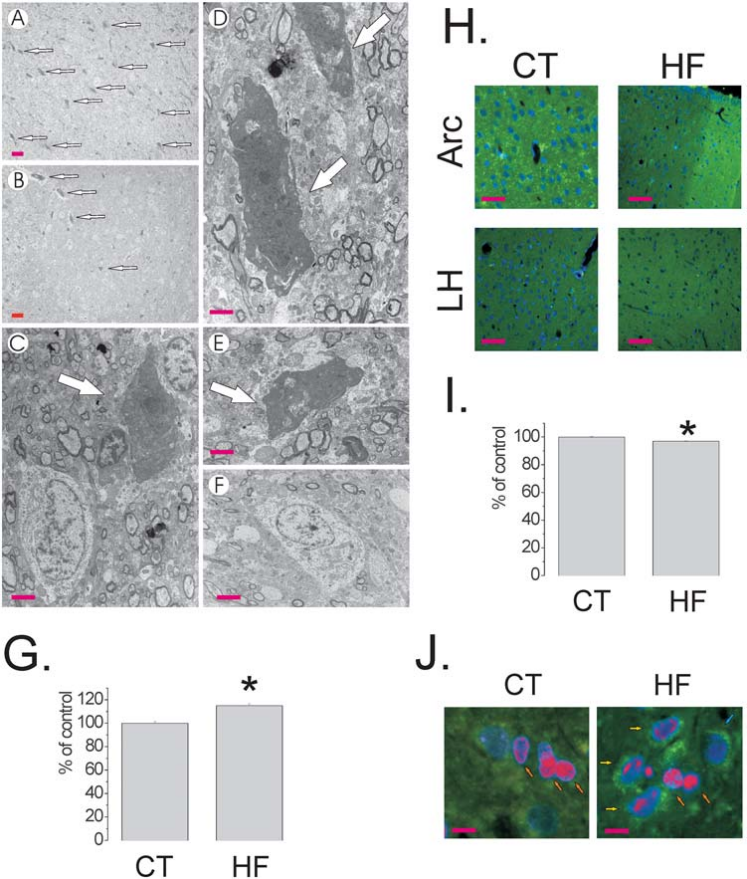
Apoptotic neurons in the hypothalamus of rats fed on high-fat diet. (A–F) Transmission electron microscopy images of typical apoptotic neurons (A–E) and a normal neuron (F) in the arcuate nucleus of Wistar rats fed on high-fat (A, C–E) and control (B, F) diets, respectively. The arrows indicate typical apoptotic neurons. (G) Apoptotic neurons were counted in low magnification fields of transmission electron microscopy analysis from the arcuate nucleus of Wistar rats fed on high-fat (HF) and control (CT) diets, the results are presented as % of CT. (H) Representative synaptophysin immunofluorescence staining of samples from arcuate (Arc) and lateral hypothalamic (LH) nuclei, from Wistar rats fed on CT or HF diets. (I) Synaptophysin positive nerve terminals were field counted and the results are presented as % of CT. (J) Representative NeuN (rhodamine) and Bax (fluorescein) double immunoflorescence staining of samples from hypothalamus of Wistar rats fed on CT and HF diets; orange arrows depict neurons without Bax expression, yellow arrows depict double positive NeuN/Bax stained neurons. A–F are representative of n = 3; magnification, *×100* (scale bar, 20 µm), A–B; and *×20,000* (scale bar, 0.2 µm), C–F. G, field counting was performed in five distinct fields from n = 3; *p<0.05 *vs.* CT. H and J are representative of n = 5; magnification, *×100* (scale bar, 40 µm), H; and *×400* (scale bar, 10 µm), J; nuclei are stained in blue by DAPI. I, nerve terminals were counted in120 distinct fields (*×200* magnification) from n = 5; *p<0.05 *vs.* CT.

Initially, we evaluated the effect of the HF diet on the induction of apoptosis in hypothalamic cells. Male Wistar rats were fed control (4% saturated fat, 15.8 kJ/g) or HF (36% saturated fat, 24.5 kJ/g) diets from the 8^th^ to 16^th^ week of life. The HF diet produced a 36±7% (69±3 *vs.* 94±4 g) increase in body mass, as compared to control (p<0.05) (Fig. 1A), which was accompanied by functional resistance to icv-injected leptin and insulin, as determined by the capacity of the hormones to inhibit 12-h spontaneous food intake [leptin, 55±8% and 21±5% inhibition in control and HF, respectively (p<0.05); insulin, 41±6% and 18±3% in control and HF, respectively (p<0.05)] (Fig. 1B–C). In addition, the HF diet led to an increased hypothalamic expression of the inflammatory cytokines TNF-α, IL-1β and IL-6, of proteins involved in inflammatory signal transduction, such as SOCS3, pJNK and pIKK (Fig. 1D), and also of a marker of glial cell activation, F4/80 (Fig. 1E). Using a real-time PCR array, we evaluated the quantitative expressions of 84 apoptosis-related genes in the hypothalamus. The HF diet modulated the expressions of 57% of the analyzed targets, suggesting a potent apoptosis/survival regulatory effect. Caspase-6, caspase-8, FADD and TNF-α receptor were some of the pro-apoptotic genes undergoing the greatest increase in the hypothalamus of HF rats, while members of the Bcl and Traf family were some of the anti-apoptotic genes modulated by the HF diet (complete results are shown in Tables 2 and 3, depicting the significantly modulated pro- and anti-apoptotic genes, respectively). Next, employing the TUNEL method, a significant increase in apoptotic cells was mapped predominantly to the arcuate and lateral hypothalamic nuclei of HF rats [arcuate, 1.9±0.1 *vs.* 14.1±0.4% TUNEL positive cells/field for control and HF, respectively (p<0.05); lateral hypothalamus, 2.1±0.1 *vs.* 15.2±0.5% TUNEL positive cells/field for control and HF, respectively (p<0.05)] (Fig. 2). Most of the apoptotic cells were neurons, as revealed by transmission electron microscopy (TEM) (Fig. 3A–F). Field counting in semi-thin sections of TEM revealed a 15.0±1.5% (p<0.05) increase in the number of apoptotic cells in HF rats (Fig. 3G), while synaptophysin positive nerve terminals (Fig. 3H) were 3.0±0.02% reduced in HF, as compared to controls (120 observations, p<0.05) (Fig. 3I), suggesting a loss of neurons and synaptic inputs. In addition, the preferential targeting of neurons was further confirmed by immunofluorescence double staining for Bax and the neuron specific antigen NeuN (Fig. 3J). Finally, the effect of the diet was site specific since the expression of the apoptotic marker Bax was virtually undetectable by immunohistochemistry in the frontal cortex, occipital cortex, parietal cortex and hippocampus (not shown).

**Table 2:** Pro-apoptotic genes modulated by HF diet.

Gene	Gene Bank
**Apaf-1**	**NM023979**
*Bak1*	*NM053812*
*Bcl10*	*NM031328*
**Bclaf1**	**XM214967**
*Bid*	*NM022684*
*Bid3*	*NM057130*
**Bik**	**NM053704**
**Card10**	**XM243622**
*Card6*	*XM226804*
**Casp1**	**NM012762**
**Casp3**	**NM012922**
**Casp4**	**NM053736**
**Casp6**	**NM031775**
**Casp7**	**NM022260**
**Casp8**	**NM022277**
**Casp9**	**NM031632**
**Casp12**	**NM130422**
*Dffa*	*NM053679*
**Fadd**	**NM152937**
**Faslg**	**NM012908**
**Mapk8ip**	**NM053777**
**Pycard**	**NM172322**
*Tnfrsf5*	*NM134360*
**Tnfrsf6**	**NM139194**
**Tnfrsf10**	**NM145681**
**Tp53**	**NM030989**
**Trp53bp**	**XM223012**
*Trp63*	*NM019221*

*Italic*, genes undergoing at least a 2-fold decrease in expression, as compared to control. **Bold**, genes undergoing at least a 2-fold increase in expression, as compared to control. Samples obtained from hypothalamus of three controls and three HF rats. Complete list of analyzed genes at www.superarray.com/rt_pcr_ product/HTML/PARN-012A.html.

**Table 3:** Anti-apoptotic genes modulated by HF diet.

Gene	Gene Bank
**Api5**	**XM342470**
**Bag1**	**XM216377**
**Bcl2**	**NM016993**
**Bcl2a1**	**NM133416**
**Bcl2l1**	**NM031535**
**Bcl2l11**	**NM022612**
*Birc1b*	*XM226742*
**Birc4**	**NM022231**
**Bnip**	**NM080897**
*Bnip2*	*XM217191*
**Bnip3**	**NM053420**
*Cflar*	*NM057138*
**Dad1**	**NM138910**
**Faim**	**NM080895**
**Polb**	**NM017141**
**Prdx2**	**NM017169**
*Prlr*	*NM012630*
**Prok2**	**NM138852**
**Traf1**	**AL406530**
*Traf3*	*XM343131*
**Traf4**	**XM220640**

*Italic*, genes undergoing at least a 2-fold decrease in expression, as compared to control. **Bold**, genes undergoing at least a 2-fold increase in expression, as compared to control. Samples obtained from hypothalamus of three controls and three HF rats. Complete list of analyzed genes at www.superarray.com/rt_pcr_ product/HTML/PARN-012A.html.

**Figure 4:**
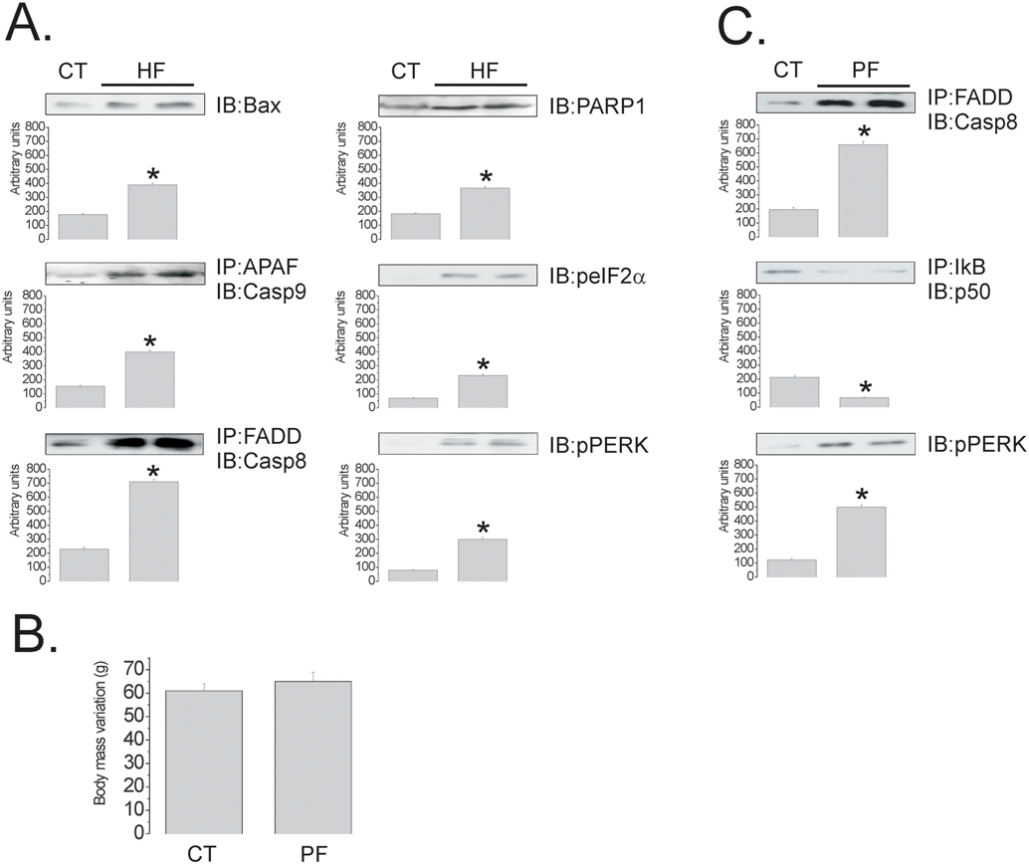
Apoptotic and endoplasmic reticulum stress markers in the hypothalamus of rats fed on high-fat diet. (A and C) Immunoblots (IB) of hypothalamic protein extracts obtained from rats fed control (CT), high-fat (HF) (A) or HF diet in pair-feeding (PF) (C); in some cases samples were submitted to immunoprecipitation (IP) prior to IB. (B) Body mass variation (g) of Wistar rats fed on CT or HF diet in pair-feeding (PF) for 8 w. In all experiments, n = 5; *p<0.05 *vs.* CT.

Proteins from both the extra- and intracellular apoptotic pathways were affected by HF diet. The hypothalamic expression of Bax and the association of APAF1 with caspase-9 (Fig. 4A), both commonly involved in intracellular apoptosis routes, and the association of FADD with caspase-8 (Fig. 4A), commonly involved in the induction of apoptosis by the extracellular route were increased in the hypothalamus of HF rats. Further evidence for apoptotic or harmful activity in the hypothalamus was shown by the increased expression of PARP1, and by the phosphorylation of proteins involved in endoplasmic reticulum stress, eIF2α and PERK (Fig. 4A).

To define whether the pro-apoptotic activity observed in the hypothalamus of HF rats was due to diet composition or caloric intake, randomly-selected rats were submitted to caloric pair-feeding for eight weeks and, at the end of the experimental period, the expression/activation of apoptosis- and endoplasmic reticulum stress-related proteins were evaluated. Pair feeding led to a similar body mass variation as control (Fig. 4B), however, the hypothalamic pro-apoptotic activity was still induced since the FADD/Caspase-8 association was increased, while the IkB/NFkBp50 association was decreased by the HF diet. In addition, the phosphorylation of PERK, a marker of ER stress, was increased by the HF diet (Fig. 4C).

**Figure 5:**
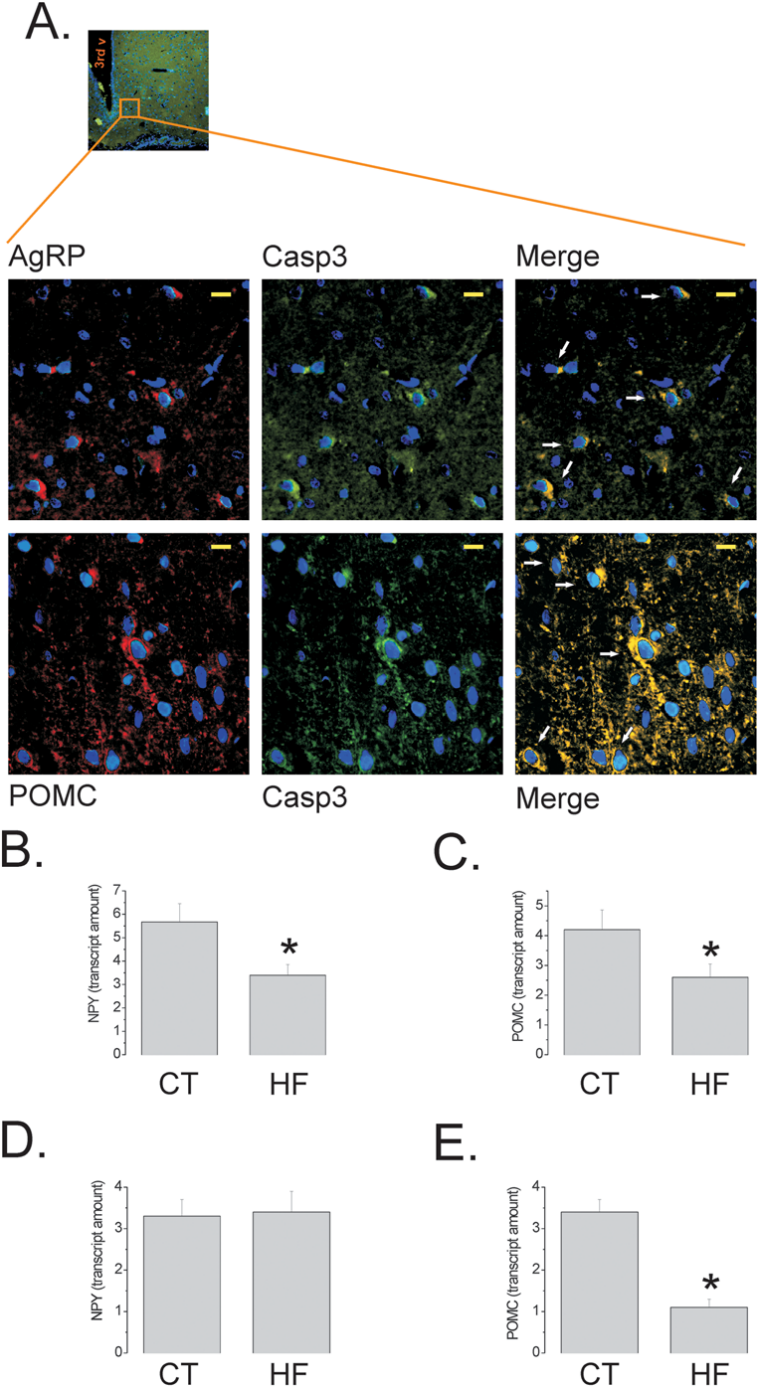
Differences in neuronal subpopulation apoptosis in diet-induced obesity. (A) Representative AgRP (rhodamine) and Caspase-3 (Casp3, fluorescein) (upper panels) or POMC (rhodamine) and Caspase-3 (Casp3, fluorescein) (lower panels) double immunoflorescence staining of samples from hypothalamus of Wistar rats fed on high-fat diet; arrows in merge depict double positive neurons; inset depicts approximate site in the arcuate nucleus that was evaluated in detail. (B–E) Real-time PCR analysis of NPY (B and D) and POMC (C and E) transcript amounts in samples obtained from the hypothalami of Wistar rats (B–C) and Swiss mice (D–E) fed on control (CT) or high-fat (HF) diets. In all experiments n = 5. In A, inset magnification, *×20* and captions magnification, *×400* (scale bar, 10 µm), nuclei are stained in blue by DAPI; 3^rd^ v, third ventricle. In B–E, *p<0.05 *vs.* CT.

To evaluate whether the apoptotic activity induced by the HF diet would affect differently the neuronal sub-populations of the arcuate nucleus, we performed double-staining immunohistochemistry in hypothalamic samples from Wistar rats and, while in control rats Caspase-3 was not detectable, in HF rats both orexigenic (AgRP, which is co-expressed with NPY) and anorexigenic (POMC) neurons expressed this apoptotic marker (Fig. 5A). This resulted in a significant and similar reduction of expression of NPY and POMC mRNA, as determined by real time PCR (Fig. 5B–C), suggesting that both subpopulations of neurons were equally affected by apoptosis. Since the obesity phenotype exhibited by Wistar rats is not extreme we evaluated the expression of the neurotransmitters in the hypothalamus of diet-induced obese Swiss mice, which develop a much more pronounced obese phenotype. As shown in Figures 5D–E, in this strain, the consumption of HF diet led to a significant reduction of POMC, but not of NPY expression.

**Figure 6:**
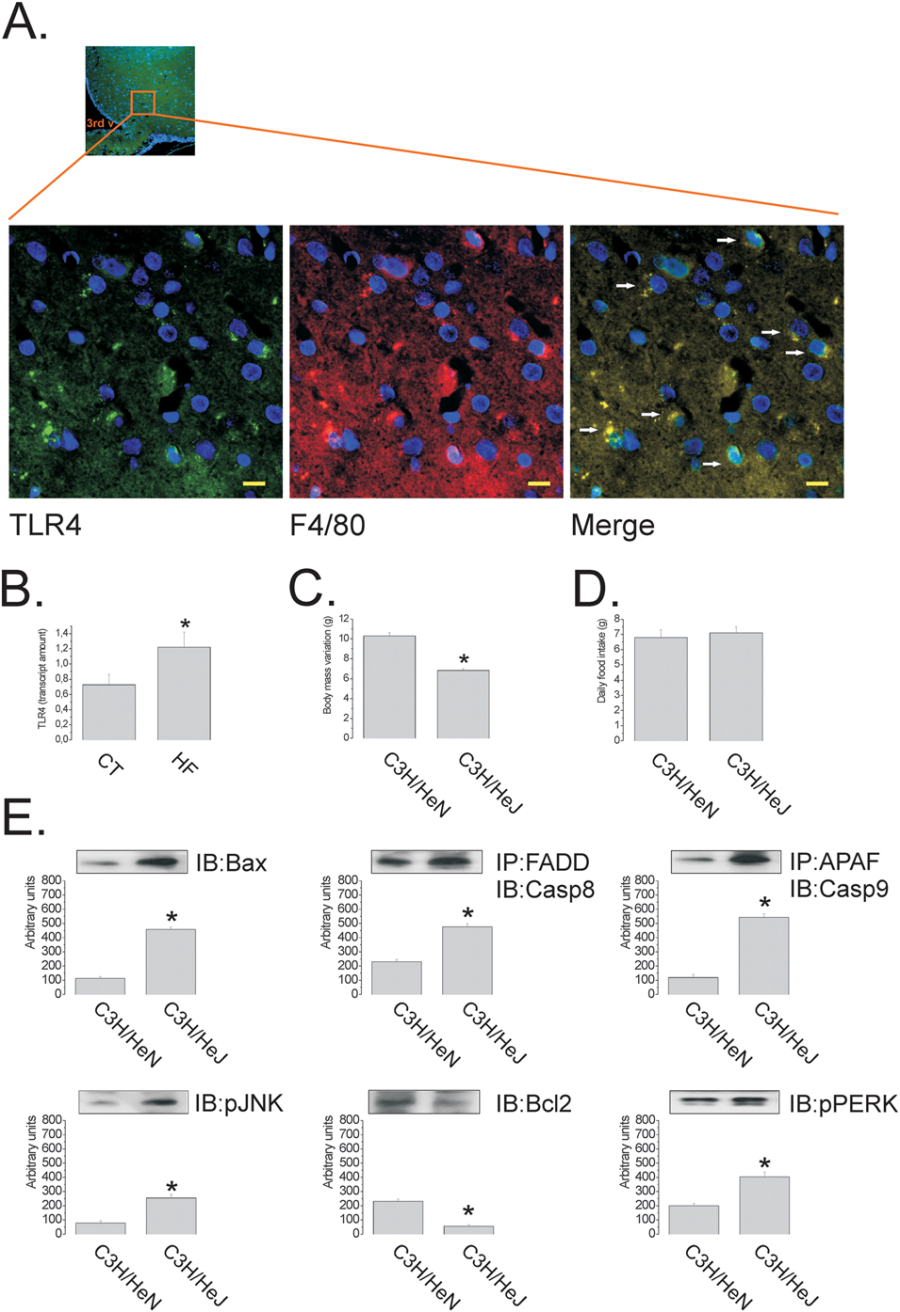
TLR4 protects against diet-induced apoptosis of hypothalamic neurons. (A) Representative TLR4 (rhodamine) and F4/80 (fluorescein) double immunoflorescence staining of samples from hypothalamus of Wistar rats; arrows in merge depict double positive cells; inset depicts approximate site in the arcuate nucleus that was evaluated in detail. (B) Real-time PCR analysis of TLR4 transcript amount in samples obtained from the hypothalami of Wistar rats fed on control (CT) or high-fat (HF) diets. (C) Body mass variation (g) of C3H/HeN and C3H/HeJ mice fed on HF diet for 8 w. (D) Mean daily food intake (g) of C3H/HeN and C3H/HeJ mice fed on HF diet. (E) Immunoblots (IB) of hypothalamic protein extracts obtained from C3H/HeN and C3H/HeJ mice fed on HF diet; in some cases samples were submitted to immunoprecipitation (IP) prior to IB. In all experiments n = 5. In A, inset magnification, *×20* and captions magnification, *×400* (scale bar, 10 µm), nuclei are stained in blue by DAPI; 3^rd^ v, third ventricle. B, *p<0.05 *vs.* CT; in C and E, *p<0.05 *vs.* C3H/HeN.

In the last part of the study, we tested the hypothesis that TLR4 could be one of the targets for dietary fats, driving or enhancing the pro-apoptotic stimulus imposed by this environmental factor in the hypothalamus. TLR4 is expressed predominantly in F4/80 positive cells of the hypothalamus (Fig. 6A) and its expression is significantly enhanced in rats fed on HF diet (Fig. 6B). Mice homozygous for the TLR4 loss-of-function mutation (C3H/HeJ) were fed on HF for 8 w and evaluated for the expression of apoptotic proteins. After 8 w on HF diet, mutant mice gained significantly less (31±4%, p<0.05) body mass than controls (C3H/HeN) (Fig. 6C), in spite of a similar food intake (Fig. 6D). However, surprisingly, the expression of Bax and the association of APAF1 with caspase-9 were increased, while the expression of Bcl2 was decreased in the hypothalamus of C3H/HeJ (Fig. 6E); in addition, the association of FADD with caspase-8 was increased (Fig. 6E). The induction of this pro-apoptotic activity was accompanied by the increased expression of markers of endoplasmic reticulum stress, pJNK and pPERK (Fig. 6E), reinforcing the worsening of the harmful effects of the HF diet in animals lacking a functional TLR4.

## Discussion

Increased consumption of dietary fats is regarded as one of the most important environmental factors predisposing to obesity in modern societies [16]. The ability of dietary fats to promote body mass gain is not exclusively due to its energetic value since prolonged caloric pair-feeding retains most of the obesogenic potential of different high-fat diets [3], [17]. Therefore, elucidating the complete mechanisms involved in high fat diet-induced obesity is of major importance to understand the pathophysiology of most cases of human obesity.

Considerable advance in this field was provided by the recent demonstration of the activation of an inflammatory response in the hypothalamus of animal models of diet-induced obesity [3], [5], [18], [19]. According to these studies, the consumption of a high-fat diet activates the expression or the activity of inflammatory responsive proteins such as SOCS3, IKK, JNK and PTP1B which impair leptin and insulin signaling in the hypothalamus, thus, disrupting the main satietogenic and adipostatic routes that maintain a stable body mass. The importance of such a mechanism is further illustrated by the fact that both genetic and pharmacological inhibition of inflammatory signaling in the hypothalamus can reverse or prevent the installation of diet-induced obesity [3], [5], [18], [19].

A rather frequent outcome of the activation of inflammatory signal transduction is the induction of pro-apoptotic signaling [7]. Cytokines such as TNF-α and IL-1β, which are highly expressed in the hypothalami of rodents fed on a high-fat diet [3], can induce apoptosis of different cell types [8]. As an example of this, in a recent study we observed that, upon icv treatment, TNF-α activates apoptotic signaling in the hypothalamus [20]. Therefore, in the first part of the study, we explored the pro-apoptotic potential of the high-fat diet evaluating the expressions of 84 apoptosis related genes by real-time PCR array. First, we showed that the diet protocol herein employed was capable of inducing the activation of markers of inflammation and resistance to leptin and insulin anorexigenic activity in the hypothalamus, as previously demonstrated in other studies [3], [5].

The effect of the diet upon the expressions of pro- and anti-apoptotic genes was remarkable. The modulation of 57% of the targets, including proteins involved in both pro- and anti-apoptotic activity, suggests that the fat-rich diet indeed has a damaging effect. As observed in other experimental settings, we suspect that the activation of some anti-apoptotic proteins provides a transient protection against the harmful effects of the diet [21], [22]. However, as shown by distinct methods, ranging from TUNEL to transmission electron microscopy, in spite of the presence of anti-apoptotic activity, apoptosis was significantly increased in the hypothalamus of the HF rats. This was an anatomical- and cell-specific phenomenon since it was detected predominantly in the hypothalamus and affected mostly neurons.

Interestingly, in Wistar rats, which develop a certain degree of obesity and do not become diabetic, the diet produced a similar reduction in the expressions of orexigenic and anorexigenic neurotransmitters, suggesting that apoptosis was evenly distributed among neuronal sub-populations. However, in Swiss mice, which are genetically related to the diabetes prone AKR mouse [23] and, likewise, display an outstanding propensity to obesity and diabetes [24], [25], the level of POMC was significantly reduced as compared to control, suggesting that this subpopulation of neurons was predominantly targeted. Although we have no current mechanistic explanation for this phenomenon, we suspect that, by targeting different subpopulations of neurons, diet-induced hypothalamic apoptosis leads to an imbalance in orexigenic *vs.* anorexigenic neurons in Swiss mice, but not in Wistar rats, therefore favoring body mass gain only in the mice. Two recent studies have provided strong evidence to suggest that changes in the numbers of certain types of hypothalamic neurons may have an impact on the control of body adiposity. First, Ryu and colleagues [10] reported that arcuate nucleus neurodegeneration caused by the depletion of Ubb, a protein involved in the production of ubiquitin, can affect energy homeostasis and lead to obesity. In addition, Kokoeva and coworkers [11] showed that hypothalamic neurogenesis induced by CNTF explains much of the sustained weight-reducing effect of this protein, a phenomenon that can be avoided by the use of an anti-mitotic agent. Therefore, we believe that, depending on genetic background and on different environmental factors, changes in neurogenesis and survival rates of hypothalamic neurons can have an impact on body adiposity.

Similarly to the effect of high-fat diets on body mass gain, here we show that activating apoptotic proteins in the hypothalamus is a property of the diet composition and not of the caloric intake. Caloric pair-feeding retained most of the pro-apoptotic activity of *ad libitum* feeding.

Recent data from ours and other groups have shown that inflammatory signaling through TLR4 plays an important role in diet-induced insulin resistance and diabetes [26], [27]. TLRs are highly-conserved members of the interleukin-1 receptor superfamily that respond to microbial signature motifs, leading to the activation of innate immune responses [28]. Some members of the TLR family are known to recognize lipid-containing motifs, such as lipopolysaccharides (LPS), which are ligands for TLR4. Whenever active, signal transduction through TLR4 leads to the coordinated induction of cytokine and other immune related genes expression [28], [29].

When we tested the hypothesis that TLR4 could mediate some of the pro-apoptotic effects of the HF diet, we obtained data that, surprisingly suggested that the presence of this receptor may be protective, rather than harmful, for cells.

Although at first sight this data may seem unexpected, recent studies have provided evidence for a protective role for TLRs in the central nervous system [30]. In many conditions, TLRs are known to mediate detrimental signals, favoring the progression of inflammatory diseases of the brain. However, an appropriately-controlled TLR activity is also important for preserving the structure and function of neural tissues exposed to harmful conditions [30]. In two previous studies the loss of TLR4 function protected against inflammation and insulin or leptin resistance [26], [27], however, on a long run, at least one study reported that in the absence of TLR4 signaling, body adiposity would increase upon high-fat diet consumption [27]. Thus, it is likely that, in diet-induced inflammation of the hypothalamus, TLR4 exerts a dual function, on one side activating pro-inflammatory pathways that play a central role in the development of resistance to leptin and insulin, and on the other side restraining further damage by controlling the apoptotic activity.

As an outcome of the present study, it will be important to investigate the proposed dual role for TLR4 in the hypothalamus, participating in the balance between inflammation and cell survival. In addition, as recent studies have shown that insulin and leptin resistance in extra-hypothalamic brain regions may relate to neurological disorders, such as Alzheimer's disease and depression [31], [32], it will be exiting to evaluate the participation of TLRs and high-fat feeding in these contexts.
