# Expression of Concern: High-Fat Diet Induces Apoptosis of Hypothalamic Neurons

**DOI:** 10.1371/journal.pone.0292912

**Published:** 2023-10-10

**Authors:** The PLOS ONE Editors

Following the publication of this article [1], concerns were raised regarding the results presented in Fig 2. Specifically, the Fig 2A CT LH panel appears to partially overlap with the Fig 2A HF LH panel, despite being used to represent different experimental conditions.

The corresponding author stated that the original data underlying the published results are no longer available. In absence of these data, the concern with Fig 2 cannot be resolved and the Fig 2 results should be interpreted with caution.

In light of this the *PLOS ONE* Editors issue this Expression of Concern to notify readers of the above concerns.
